# Supplementary material for: Transcriptomic Analysis of the Host Response to Mild and Severe CTV Strains in Naturally Infected Citrus sinensis Orchards
Source: Int J Mol Sci. 2022 Feb 23;23(5):2435. doi: 10.3390/ijms23052435 (PMC8910659; doi:10.3390/ijms23052435)
Supplement: Supplementary file 1 [file ijms-23-02435-s001.zip › Supplementary Material Ramirez-Pool et al 2022.pdf]

Supplementary material
Ramírez-Pool et al., 2022

Supplementary tables

Table S1. Sample collecting sites of sweet orange trees showing CTV symptoms in Veracruz, Mexico.

Sample	Latitude (°N)	Longitude (°W)	Symptoms
Sample 1	20.66846	-97.37957	Mild
Sample 2	20.66846	-97.37926	Mild
Sample 3	20.66861	-97.37892	Asymptomatic
Sample 4	20.67187	-97.38067	Mild
Sample 5	20.67201	-97.3797	Mild
Sample 6	20.67158	-97.38015	Asymptomatic
Sample 7	20.71471	-97.38503	Severe
Sample 8	20.71465	-97.38509	Severe
Sample 9	20.71504	-97.38496	Asymptomatic
Sample 10	20.7391	-97.38067	Severe
Sample 11	20.71392	-97.38067	Severe
Sample 12	20.70333	-97.21888	Asymptomatic
Sample 13	20.70336	-97.21905	Mild
Sample 14	20.69525	-97.29107	Asymptomatic
Sample 15	20.69546	-97.2908	Asymptomatic
Sample 16	20.66859	-97.37899	Asymptomatic
Sample 17	20.71505	-97.38492	Asymptomatic
Sample 18	20.71382	-97.38017	Asymptomatic
Sample 19	20.66468	-97.38785	Severe
Sample 20	20.66958	-97.38049	Mild
Sample 21	20.65780	-97.38913	Mild

Table S2. Summary of sequence reads and unigenes.

A) Raw data read and high-quality read numbers, after using Quality Control Python Script.

**Condition**	**Raw data reads**			**High quality reads**		
	Forward (R1)	Reverse (R2)	Total	Forward (R1)	Reverse (R2)	Total
Asymptomatic	11,554,453	11,554,453	23,108,906	11,284,456	11,284,456	22,568,912
Severe CTV strain	10,497,308	10,497,308	20,994,616	10,275,130	10,275,130	20,550,260
Mild CTV strain	10,352,061	10,352,061	20,704,122	10,131,387	10,131,387	20,262,774
			64,807,644			63,381,946

B) Longer reads obtained by merging paired-end reads with overlapping regions and unmerged paired-end reads using SeqPrep.

**Condition**	**Merged paired-end reads**	**Unmerged paired-end reads**	
		Forward (R1)	Reverse (R2)
Asymptomatic	8,464,587	2,690,443	2,690,443
Severe CTV strain	7,827,596	2,301,768	2,301,768
Mild CTV strain	7,660,822	2,339,494	2,339,494
	23,953,005	14,663,410	

C) Clean unigenes obtained with different bioinformatic programs.

**Project**	**Trinity**	**DeconSeq**	**AlignWise**	**BlastClust**
Transcriptome assembly *C. sinensis* (Veracruz)	124,794	124,215	91,548	83,672

Table S3. Genes related to signaling and disease resistance in response to CTV infection.

Arabidopsis gene ID	*C. sinensis* gene ID	Description[1]	log_2 fold change	
			Mild infection	Severe infection
AT3G14470	XP_006466045.1	NB-ARC domain-containing disease resistance protein.	7.1293	8.3966
AT3G47570	XP_006470403.1	Leucine-rich repeat protein kinase family protein.	7.5774	0
AT4G39090	XP_006473584.1	Rd19 cysteine proteinases, induced by desiccation. Required for RRS1-R mediated resistance against *Ralstonia solanacearum*.	4.2743	0
AT5G17680	XP_024953872.1	Disease resistance protein (TIR-NBS-LRR class).	5.4683	0
AT1G06840	XP_006464962.1	Probable LRR receptor-like serine/threonine-protein kinase.	9.7993	0
AT1G53430	XP_024957540.1	Probable LRR receptor-like serine/threonine-protein kinase.	8.3219	0
AT4G28300	XP_015383147.2	A prion-like protein regulator of seed germination undergoes hydration-dependent phase separation.	-8.2574	0
AT4G27190	XP_006475172.1	Disease resistance protein RPS2-like isoform X2.	7.3219	0
AT4G33720	XP_006486820.1	Pathogenesis-related protein 1-like.	5.8784	0
AT3G04720	XP_006487793.1	Pathogenesis-related protein PR-4A.	4.9185	0
AT1G20030	XP_006493553.1	Thaumatin-like protein 1b.	5.1851	-7.5925
AT1G17860	XP_006465039.1	Kunitz trypsin inhibitor 2-like.	8.7616	0
AT3G54420	NP_001275834.1	Chitinase CHI1 precursor.	12.1969	-7.3038
AT3G12500	NP_001306999.1	Endochitinase-like precursor.	7.0524	0
AT1G02800	NP_001275794.1	Acidic cellulase.	8.4263	0
AT4G39400	XP_006464515.1	Plasma membrane localized leucine-rich repeat receptor kinase involved in brassinosteroid signal transduction.	0	-8.2668
AT4G16990	XP_006479988.1	Disease resistance protein (TIR-NBS class).	-6.7460	-8.3309
AT5G18350	XP_006478367.1	Protein suppressor of npr1-1, constitutive 1-like.	0	6.4094
AT2G31880	XP_006478632.1	Leucine-rich repeat receptor-like	0	8.3923

		serine/threonine/tyrosine-protein. kinase SOBIR1.		
AT3G47110	XP_024955845.1	Putative receptor-like protein kinase.	0	8.6294
AT4G11170	XP_024949622.1	Putative disease resistance protein.	0	-7.6724
AT4G08850	XP_024953388.1	Probable leucine-rich repeat receptor-like protein kinase.	0	7.8202

[1] Source: https://www.arabidopsis.org/
https://www.ncbi.nlm.nih.gov/

Table S4. Genes related to DNA replication, repair and translation in response to CTV infection.

Arabidopsis ID	*C. sinensis* ID	Description[1]	log_2 fold change
**Downregulated genes in mild CTV-infected plants**			
DNA replication/genome integrity/DNA repair			
AT2G24490	XP_006483593.1	Replication protein A 32 kDa subunit A	-7.3399
AT5G13820	XP_006486468.1	Telomere repeat-binding protein 4 isoform X2	-9.5962
AT1G00990	XP_006490999.1	FEN1 flap endonuclease	-7.3038
AT3G28030	XP_006470382.1	DNA repair protein UVH3 isoform X4	-8.2192
AT5G11580	XP_006476033.1	Ultraviolet-B receptor UVR8 isoform X1	-6.8948
AT5G64420	XP_015388269.1	Myb-binding protein 1A	-8.2854
AT2G22720	XP_006466376.1	Protein SPT2	-8.5236
AT2G27170	XP_006487808.1	Structural maintenance of chromosomes protein 3	-7.5774
Translation			
AT1G17080	XP_006477943.1	60S ribosomal protein L18a-like protein isoform X1	-7.6221
AT1G76810	XP_006473041.1	Eukaryotic translation initiation factor 5B	-9.7830
AT2G04520	XP_006469570.1	Eukaryotic translation initiation factor 1A	-9.4429
AT2G31610	XP_006466354.1	40S ribosomal protein S3-3	-7.8642
AT3G01740	XP_024951194.1	54S ribosomal protein L37, mitochondria	-7.4594
AT3G25520	XP_006492784.1	60S ribosomal protein L5	-7.7549
AT3G44590	XP_006471376.1	60S acidic ribosomal protein P2A-like	-7.6935
AT3G55280	XP_006486050.1	60S ribosomal protein L23a	-8.1849
AT3G60240	XP_006464388.1	Eukaryotic translation initiation factor 4G-like	-7.7279
AT4G11420	XP_006472155.1	Eukaryotic translation initiation factor 3 subunit A	-8.6257
AT4G33740	XP_024952836.1	Ribosomal RNA processing protein 1 homolog	-8.1241
AT5G38640	XP_006494252.1	Translation initiation factor eIF-2B subunit delta isoform X	-7.4998
**Upregulated genes in mild CTV-infected plants**			
DNA replication/genome integrity/DNA repair			
AT4G11670	XP_006477423.1	Protein unc-13 homolog isoform X1	7.5622
AT5G22330	XP_006482057.1	RuvB-like protein 1	7.8455
AT1G20720.1	XP_006473806.1	RAD3-like DNA-binding helicase protein	8.2900

Translation			
AT5G19510	XP_006470501.1	Elongation factor 1-beta 2	4.9619
AT2G21580	XP_006487218.1	40S ribosomal protein S25-2	7.3750
**Downregulated genes in severe CTV-infected plants**			
DNA replication/genome integrity/DNA repair			
AT2G24490	XP_006483593.1	Replication protein A 32 kDa subunit A	-7.3399
AT5G63920	XP_006465105.1	DNA topoisomerase 3-alpha isoform X1	-7.4346
AT5G55310	XP_024953709.1	DNA topoisomerase 1 beta-like isoform X1	-8.2046
AT1G17980	XP_006493030.1	Nuclear poly(A) polymerase 1 isoform X1	-7.4263
AT4G05420	XP_015382568.1	DNA damage-binding protein 1a isoform X2	-7.2095
AT5G64420	XP_015388269.1	Myb-binding protein 1A	-8.2854
AT5G11310	XP_024953685.1	Ultraviolet-B receptor UVR8 isoform X2	-7.5999
AT5G15860	XP_024949988.1	Sister chromatid cohesion 1 protein 4 isoform X2	-7.2854
Translation			
AT2G44065	XP_024951250.1	Ribosomal protein L2 family	-7.9248
AT5G24510	XP_006491363.1	60S acidic ribosomal protein P1-like	-7.1799
AT2G39390	XP_006486009.1	60S ribosomal protein L35	-7.0768
AT2G27710	XP_006471376.1	60S acidic ribosomal protein P2A-like	-7.6935
AT3G55280	XP_006486050.1	60S ribosomal protein L23a	-8.1849
AT5G39740	XP_006492784.1	60S ribosomal protein L5	-6.7549
AT1G17080	XP_006477943.1	60S ribosomal protein L18a-like protein isoform X1	-7.6221
AT3G59540	XP_006480494.1	60S ribosomal protein L38	-7.3038
AT1G07940	XP_006485903.1	Elongation factor 1-alpha	-4.4175
AT5G60390	XP_015388818.1	Elongation factor 1-alpha	-9.4878
AT1G76810	XP_006473041.1	Eukaryotic translation initiation factor 5B	-9.7830
AT3G60240	XP_006464388.1	Eukaryotic translation initiation factor 4G-like	-7.7279
AT2G04520	XP_006469570.1	Eukaryotic translation initiation factor 1A	-9.4429
AT5G24510	XP_006491363.1	60S acidic ribosomal protein P1-like	-7.1799
AT1G26910	XP_006486128.1	60S ribosomal protein L10	-7.7616
**Upregulated genes in severe CTV-infected plants**			
DNA replication/genome integrity/DNA repair			
AT5G13820	XP_006486468.1	Telomere repeat-binding protein 4 isoform X2	7.4179
AT5G59910	XP_006488268.1	Histone H2B.3	7.1085
Translation			
AT2G21580	XP_006487218.1	40S ribosomal protein S25-2	7.1997
AT3G59670	XP_006466516.1	Elongation factor LOC102619044	7.3663

AT3G07300	XP_006470847.1	Translation initiation factor eIF-2B subunit β	7.2095

[1] Source: https://www.arabidopsis.org/
https://www.ncbi.nlm.nih.gov/

Table S5. Genes involved in PTGS, epigenetic regulation and RNA processing in response to CTV infection.

**Arabidopsis ID**	***C. sinensis* ID**	**Description[1]**	**log_2 fold change**	
			Mild infection	Severe infection
AT3G27860	XP_006470311.1	Tudor/PWWP/MBT superfamily protein.	7.9542	0
AT1G48410	XP_006478120.1	Protein argonaute 1 isoform X2.	-4.5397	-9.4939
AT2G13370	XP_006470733.1	Protein argonaute 4-like.	-7.4263	7.9307
AT5G52230	XP_006490367.1	Methyl-CpG-binding domain-containing protein 13 isoform X1.	-7.000	-8.5850
AT5G14170	XP_006470053.1	SWI/SNF complex component SNF12 homolog.	-8.1749	0
AT2G28600	XP_006492170.1	DEAD-box ATP-dependent RNA helicase 5 isoform X1.	7.7279	0
AT1G27900	XP_006484063.1	Probable pre-mRNA-splicing factor ATP-dependent RNA helicase DEAH4 isoform X1.	-8.1599	0
AT3G22330	XP_015386756.1	DEAD-box ATP-dependent RNA helicase 53, mitochondrial-like.	-8.1293	0
---	XP_024953685.1	Regulator of chromosome condensation (RCC1) family protein.	0	-7.5999
---	XP_024948665.1	Histone-lysine N-methyltransferase SUVR5 isoform X2.	0	-7.2761
AT4G16310	XP_015384332.1	Lysine-specific histone demethylase 1 homolog 3 isoform X3.	0	-7.1699
AT3G26850	XP_015385461.1	Histone-lysine N-methyltransferase; zinc finger CCCH domain-containing protein 55-like isoform X3.	0	7.2384
AT1G20670	XP_024958045.1	DNA-binding bromodomain-containing protein.	0	-7.9887
AT2G27040	XP_006470434.1	Protein argonaute 4-like.	-7.4263	7.9307
AT5G23570	XP_024952165.1	Protein suppressor of gene silencing 3.	0	8.3354

[1] Source: https://www.arabidopsis.org/
https://www.ncbi.nlm.nih.gov/

Table S6. Primers used to validate the RNA-Seq experiment by RT-qPCR.

Primer ID	Sequence 5′ → 3′
XP_006475507.1_FW	GCATGTGATGTTGCTCGAAG
XP_006475507.1_RV	GCAAGCTTGGCAAGGTAATC
XP_006470053.1_FW	TCCCTTGATGGAGGTTCTTG
XP_006470053.1_RV	GGTGCTGCAATTTTCTAGCC
XP_024949213.1_FW	CGTGGTCGAACTGATTGTTG
XP_024949213.1_RV	ACACTTTCCATCTCCCCAAG
XP_006490176.1_FW	TGTGGAAGATGGACCAAACC
XP_006490176.1_RV	AGCAACCTCCCCGTTTTTAC
XP_024953872.1_FW	CGTATGATTGGGATCTGTGG
XP_024953872.1_RV	GATCAGACCGCCTTTTTCTG
XP_006470311.1_FW	ATGTTGGTGCAGAGCATGAG
XP_006470311.1_RV	GAGCGGATTCCAGAAATGAC
XP_006483041.1_FW	CGCTGAGTGCAATTATGTGG
XP_006483041.1_RV	ACGATATTCGGGAGAGCAAC
XP_006485913.1_FW	ATACGGAAACGTGGGAACAG
XP_006485913.1_RV	TTTCTCTTGGGCTTGTCCTG
XP_006468074.1_FW	ATTTCTGCAGAGGGAGATGC
XP_006468074.1_RV	AGGTCTCCCATTTGGTTGTG
XP_006471773.1_FW	ATACCCCTTGCCACATCAAG
XP_006471773.1_RV	TGTTACCCGTCCTCCAAATC
XP_024949989.1_FW	GGCTTCTGTTGATGTGCAAG
XP_024949989.1_RV	TACTGGCAGTTGCATTGTCC
XP_006479423.1_FW	TGGAAATCTCAGCGGAAGAC
XP_006479423.1_RV	ATATCACGAGCTGGGGTTTG
CsACT-F [1]	ACTTCGTCTTGACCTTGCTG
CsACT-F [1]	TCAAGAGCGATGTAAGCCAG

[1] Mafra et al., 2012

Supplementary figures

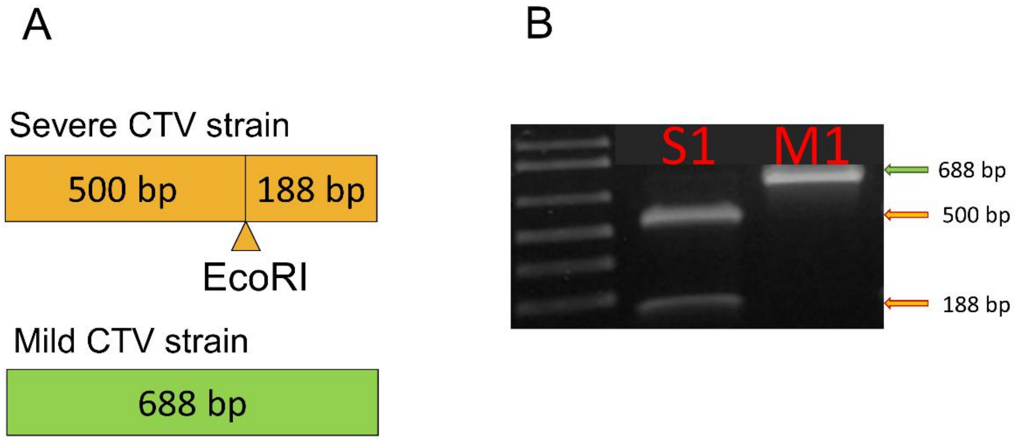

Figure S1. Identification of severe or mild CTV strain by digestion with EcoRI restriction enzyme. A) Schematic representation of the single nucleotide polymorphism (SNP) in coat protein (CP) gene of 688 bp of a severe CTV strain with an EcoRI restriction site at 500 bp (upper panel), and a mild CTV strain with no restriction site (lower panel). B) Agarose gel electrophoresis of the RT-PCR from CP gene of a severe CTV strain (S1) and a mild CTV strain (M1). 1Kb Plus DNA ladder marker (Invitrogen) was used.

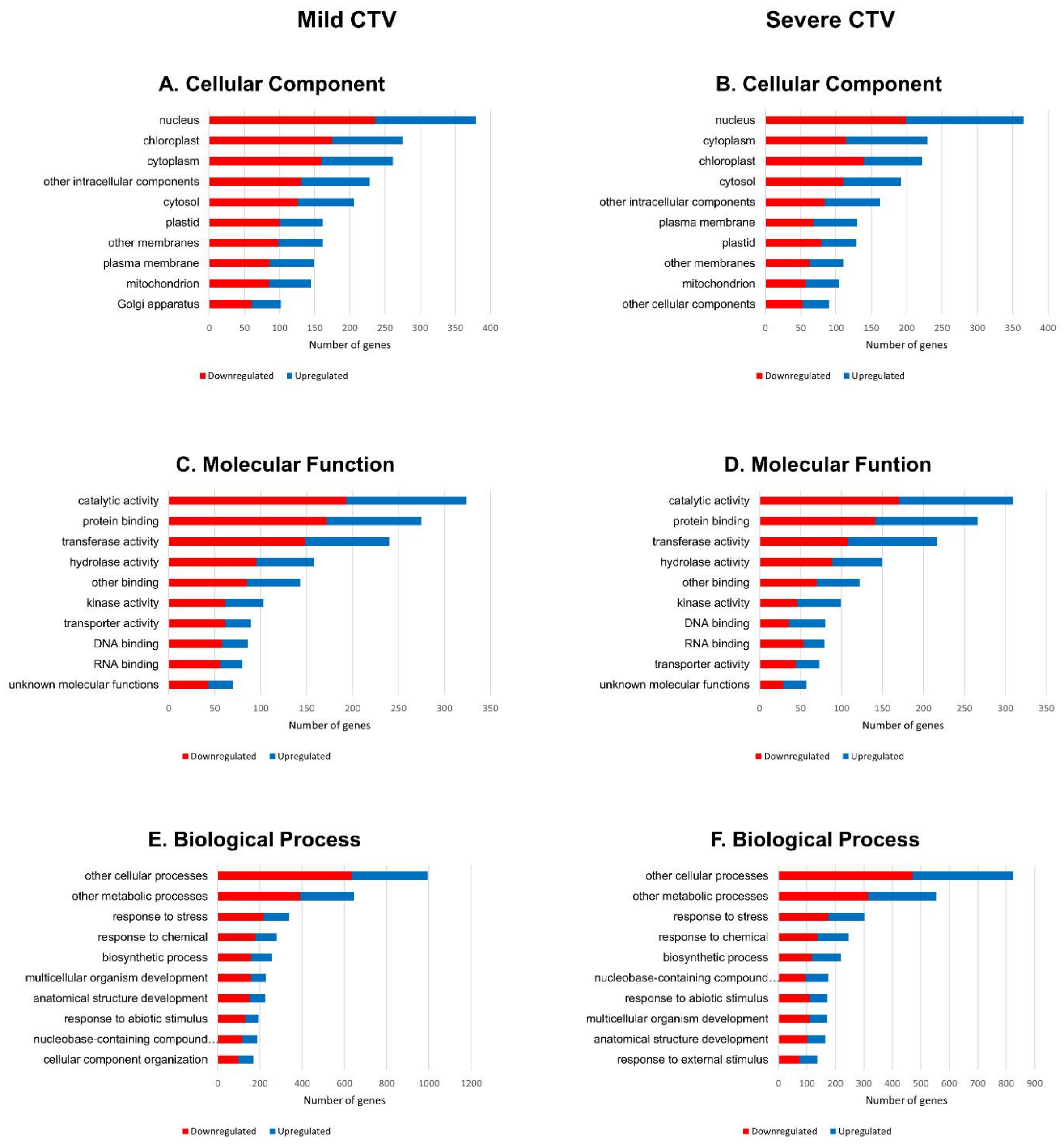

Figure S2. Classification of differentially expressed transcripts in GO terms of biological functions in plants infected with mild (left) and severe (right) CTV strains. (A) Cellular component; (B) Molecular function; and (C) Biological process. Red bars represent downregulated genes, blue bars represent upregulated genes. Number of genes (hits) are shown on the x-axis.

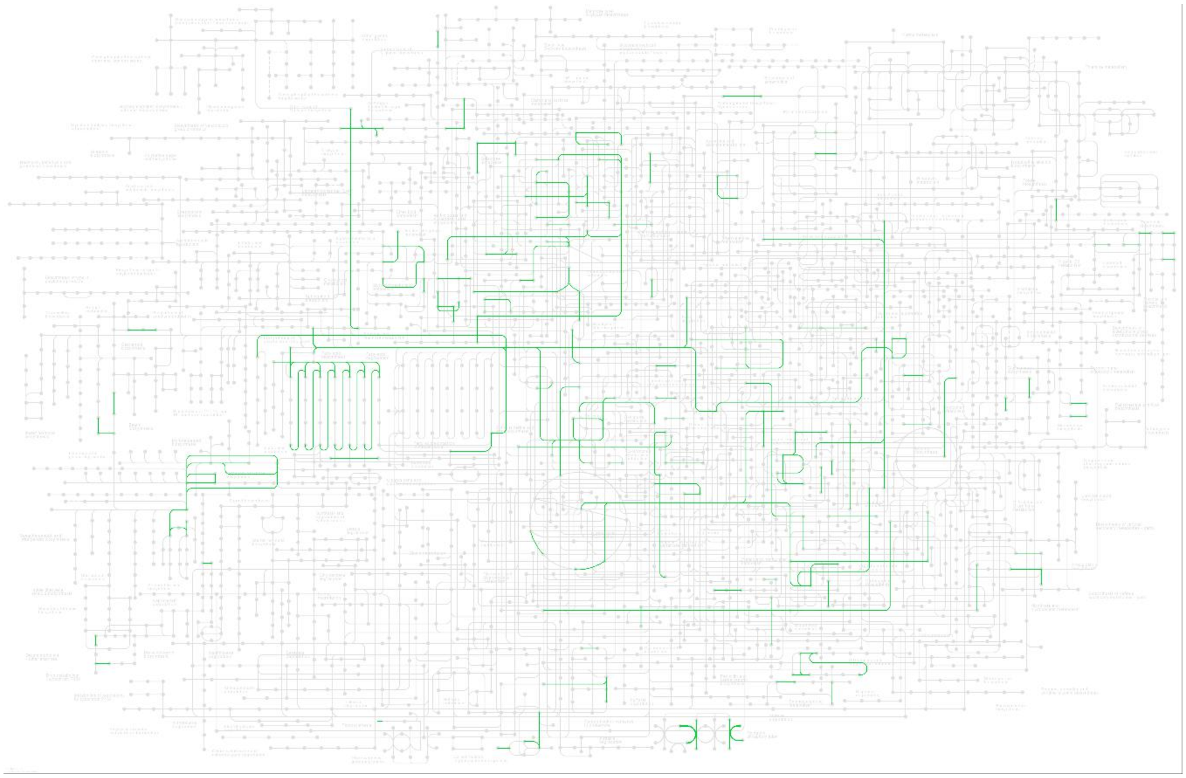

Figure S3. Metabolic pathways affected in mildly CTV-infected plants. Green lines indicate differentially regulated pathways.

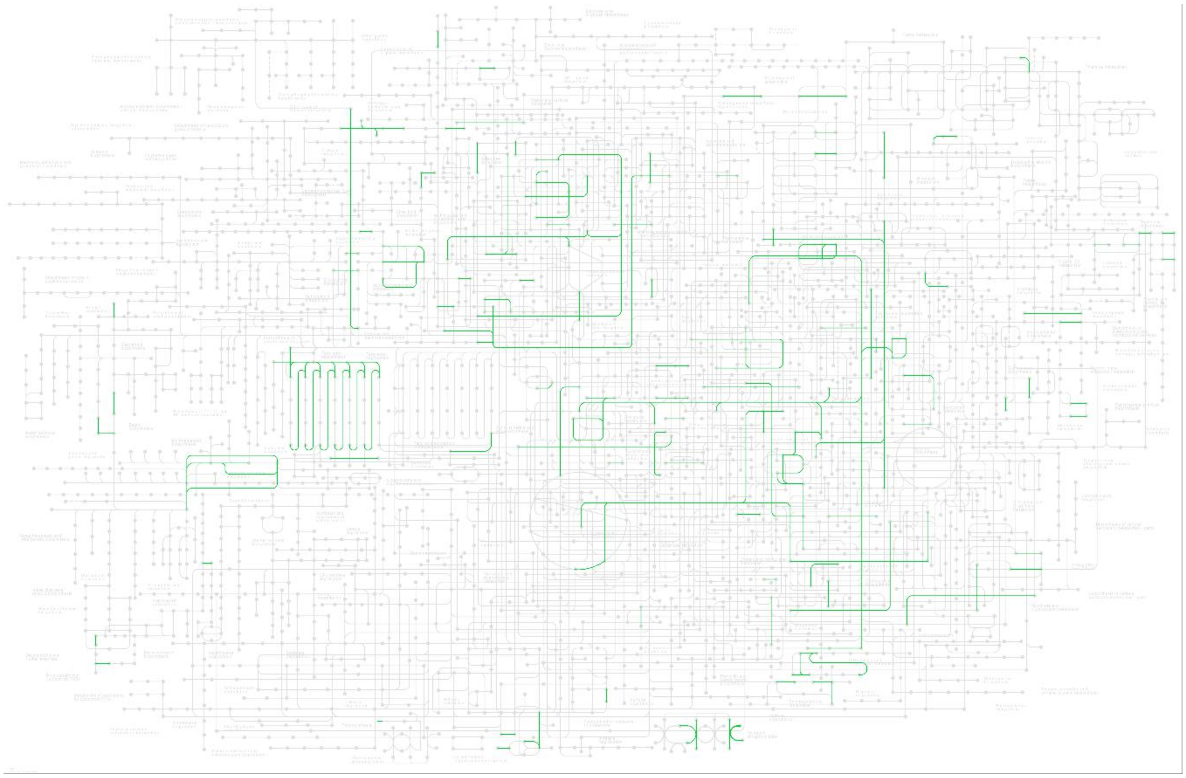

Figure S4. Metabolic pathways affected in severely CTV-infected plants. Green lines indicate differentially regulated pathways.

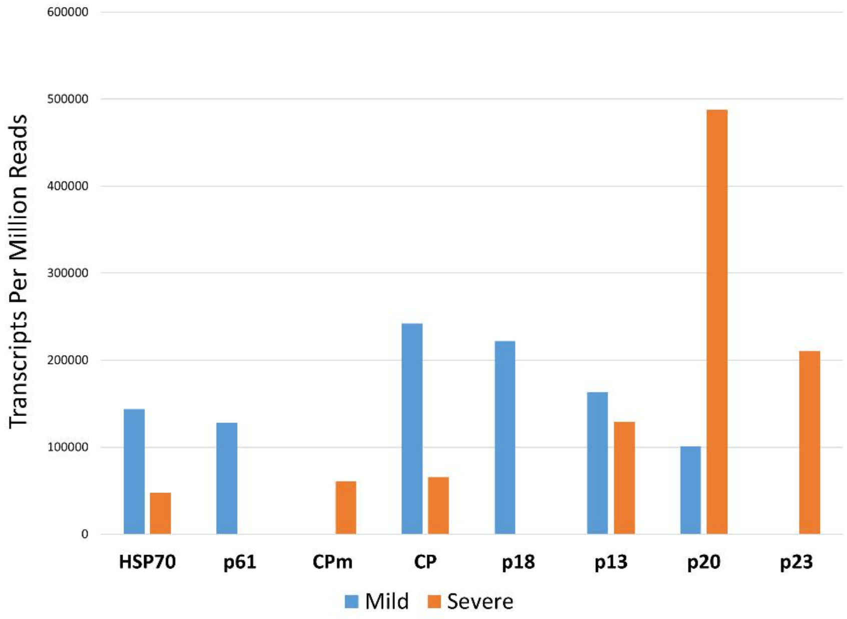

Figure S5. Comparison of viral ORF transcripts per million reads obtained in plants infected with either mild (blue) or severe (orange) CTV strains. Gene description: HSP70H, Hsp70-homolog; p61, 61-kDa protein; CPM, minor capsid protein; CP, major capsid protein; p18, 18-kDa protein; p13, 13-kDa protein; p20, 20-kDa protein/RNA silencing suppressor; p23, RNA silencing suppressor.
